# Ultrasound Evaluation of Tendinopathy in Hemophiliac Patients for the Purpose of Rehabilitation Indications

**DOI:** 10.3390/jcm12134513

**Published:** 2023-07-06

**Authors:** Danilo Donati, Paolo Spinnato, Lelia Valdrè, Lydia Piscitelli, Giuseppina Mariagrazia Farella, Enrico Pagliarulo, Maria Grazia Benedetti

**Affiliations:** 1 Physical Therapy and Rehabilitation Unit, Policlinico di Modena, 41125 Modena, Italy; 2 Clinical and Experimental Medicine PhD Program, University of Modena and Reggio Emilia, 41124 Modena, Italy; 3 Diagnostic and Interventional Radiology, IRCCS Istituto Ortopedico Rizzoli, 40134 Bologna, Italy; 4 Inherited Bleeding Disorders Unit, IRCCS AOUBO, 40138 Bologna, Italy; 5 Physical Therapy and Rehabilitation Unit, IRCCS Istituto Ortopedico Rizzoli, University of Bologna, 40134 Bologna, Italymariagrazia.benedetti@ior.it (M.G.B.)

**Keywords:** hemophilia, tendinopathy, ultrasound, rehabilitation

## Abstract

Background: Hemophilia is a inherited bleeding disorder that is characterized by intra-articular bleeding (hemarthrosis). The aim of the study was to evaluate the state of the satellite tendons of the target joints in the patient with hemophilic arthropathy and propose rehabilitation treatment with eccentric exercises. Methods: The tendons of the joints mainly affected by hemophilic arthropathy were evaluated by ultrasound. The ultrasound evaluation is associated with the use of evaluation clinical scales, such as the Hemophilia Joint Health Score (HJHS), the Functional Independence Score in Hemophilia (FISH), the Hemophilia Activity List (HAL), the DASH, the VISA-A, the VISA-P, and the VAS scale. Results: In 20 patients with hemophilic arthropathy, the thickness of the tendons that were examined was normal. In six subjects with severe joint damage, echostructural alterations were present, and signs of hyperemia and neo-vascularization were detected on color Doppler, as well as the presence of intratendinous calcifications. Conclusions: The tendons of the target joints in patients with hemophilic arthropathy are compromised by the indirect biomechanical damage caused by the joint disease, and rehabilitation treatment with eccentric exercises can be considered safe and effective in improving the tenso-elastic properties of the tendons.

## 1. Introduction

Hemophilia is a inherited bleeding disorder that is characterized by recurring bleeding episodes. The most common forms are hemophilia A, also known as a deficiency of factor VIII, and hemophilia B, factor IX deficiency. Both are hereditary genetic diseases linked to the X chromosome. A typical clinical manifestation of hemophilia is intra-articular bleeding (hemarthrosis), which affects the main joints, such as the elbow, knee, and ankle, associated with muscle hematoma [1,2]. Repeated hemarthrosis can cause degenerative arthropathy, a frequent complication in patients with moderate and severe hemophilia. Hemophilic arthropathy is a secondary form of osteoarthritis that is characterized by an ultrasound level with the presence of intra-articular blood effusion, which can cause synovitis and synovial hypertrophy and causes damage to the articular cartilage [3,4]. Ultrasound has become a first-level diagnostic method in the musculoskeletal field for the study of the joint damage associated with the pathology and any alteration affecting the periarticular structures [5,6,7]. There are also several studies in the literature about the use of ultrasound in the assessment and staging of joint damage caused by hemophilic pathology. In particular, the most used score is the “Hemophilia Early Arthropathy Detection with Ultrasound (HEAD-US)”, shared among all professionals dealing with hemophilia [8,9]. However, it is conceivable that, following an inflammatory process involving the joint, the periarticular structures, such as the tendons, may be involved because of the relationship of continuity with the capsular structures, as seen in the knee and elbow [10]. Furthermore, in the presence of chronic arthropathy with joint deformity and functional limitation, it is possible that the tendons undergo degeneration processes related to disuse or biomechanical alterations [11,12]. To our knowledge, the morphological study of tendons in hemophiliac patients has never been performed, although there is little experience on the assessment of the elasticity of tendons in patients with hemophilic arthropathy by means of elastosonography. In the studies by Cruz-Montecinos et al., greater rigidity and hysteresis of the Achilles tendon compared to healthy subjects and greater joint stiffness during evaluation of the tendon during stretching exercises were found [13,14]. These scientific data, accompanied by our clinical experience, support the importance of a greater morphological knowledge of tendon tissue, aimed at ensuring safe rehabilitative treatments, such as eccentric exercise or stretching, in order to counteract the joint stiffness and muscle hypotrophy associated with the possible presence of tendinopathy. The aim of this study was to evaluate the state of the satellite tendons of the target joints in patients with hemophilic arthropathy to search for possible tendons alterations.

## 2. Materials and Methods

### 2.1. Subjects

Patients with hemophilic arthropathy were sent by the Inherited Bleeding Disorders Unit in Bologna to the Physical and Rehabilitative Medicine Department of the Rizzoli Orthopedic Institute as part of a clinical collaboration for the rehabilitation needs of these patients. During their examinations, patients were asked to participate in the study for the ultrasound evaluation of tendons. The study was approved by the Ethics Committee of the Institute (PG no. 0010368 del 17/06/2021), and all the patients included signed the informed-consent form to participate. Patients were screened on the basis of age, between 18 and 65 years, and the presence of ultrasound signs of hemophilic arthropathy. Exclusion criteria were the presence of elbow, knee, and ankle arthroplasty; and ankle arthrodesis, knee, and ankle synovectomy. In agreement with the Ethics Committee, the study was designed as a pilot study including a number of patients so that at least 30 ultrasound examinations were performed on the target tendons of the study (10 on the biceps/triceps tendons for the elbow, 10 on the quadriceps/patellar tendons for the knee, and 10 on the Achilles tendon of the ankle). Since hemophilic pathology has a polyarticular involvement, multiple joints were evaluated in the same patient.

### 2.2. Ultrasound Assessment

Biceps and triceps brachii tendons were assessed at the elbow, quadriceps and patellar tendons at the knee, and Achilles tendons at the ankle. Tendons were assessed bilaterally in order to carry out a comparative study. A musculoskeletal radiologist with 11 years of experience (P.S.) and physiatrist expert in musculoskeletal ultrasound with 3 years of experience (D.D.) performed the US evaluations in consensus.

The ultrasound examination was performed with a linear probe with musculoskeletal presets for elbow’s tendons evaluation (7–16 MHz). The following ultrasound parameters were evaluated:

Maximum tendon thickness (anterior-posterior diameter).

For the tendon thickness, there are no reference values because it is highly variable for age, sex, and race.

Echogenicity and echostructure alteration (in a visual semi-quantitative way). The classification is reported in Table 1.

Signs of hyperemia on power color Doppler (neovascularization scale). The classification is reported in Table 1.

Presence/absence of calcifications/other tendon ultrasound findings [15].

### 2.3. Clinical–Functional Assessment

The ultrasound evaluation was associated with the administration of clinical–functional assessment scales:

The Hemophilia Joint Health Score (HJHS) is a measure of the structural damage caused by the recurrence of bleeding in the joints. For each joint (knee and ankle), the swelling, duration of swelling, muscle atrophy, axial alignment, instability, crepitus on motion, loss of flexion, loss of extension, pain, loss of strength, and gait are evaluated. The score ranges from 0 to 132, where 0 is the best joint condition and 132 is the worst [16,17].

The Hemophilia Activity List (HAL) investigates functional activities related to postural changes (sitting, getting up, and kneeling), functioning of the lower limbs (walking, running, and stairs), functioning of the upper limbs (lifting objects and buttoning a shirt), use of means of transport (car, bicycle, and bus), self-care, home activities, leisure and sports, and use of aids and/or adaptations. In total, there are 7 domains and 42 questions. The score is normalized and ranges from 0 (worst functional status) to 100 (best functional status) [18].

The Functional Independence Score for Hemophilia (FISH) investigates 8 activities: nutrition, hygiene, dressing, sitting, bending, walking, stairs, and running. The total score varies from 8 to 32, where a total score of 8 corresponds to the worst situation, with the complete inability to carry out each activity, and the maximum score of 32 corresponds to the best situation, where all the activities examined are carried out normally [19].

The Visual Analogic Scale (VAS) is a one-dimensional scale that evaluates the intensity of pain. The scale ranges from 0 to 10, with 0 being no pain and 10 being the greatest possible pain [20].

Disability of the Arm, Shoulder, and Hand (DASH) is a measure of function and symptoms in patients with any musculoskeletal alteration of the upper limbs. The questions refer to the ability to perform certain actions in the last week and the symptoms that have arisen while performing these actions. The percentage score can vary from a minimum of 0% to a maximum of 100%, where, as the score increases, there is an increase in the patient’s inability to carry out activities of daily living [21,22].

The Victorian Institute of Sports Assessment—Achilles questionnaire (VISA-A) is designed for patellar tendon pain; the questions measure and describe the main symptoms (pain and stiffness) given by yarrow tendinopathy, so as to define the degree and progression through activities of daily living (e.g., going down the stairs). The maximum score is 100, indicating someone who is completely asymptomatic. A lower score indicates more symptoms and greater limitation of physical activity. A person with Achilles tendinopathy will not score higher than 70 on the VISA-A scale [23].

The Victorian Institute of Sports Assessment—Patellar questionnaire (VISA-P) is designed for patellar tendon pain; it measures and describes the main symptoms (pain and stiffness) of patellar tendinopathy, so as to define the degree and progression through activities of daily living (e.g., going down the stairs). The maximum score is 100, indicating someone who is completely asymptomatic. A lower score indicates more symptoms and greater limitation of physical activity. A person who has patellar tendinopathy will not score higher than 60 on the VISA-P scale [24].

### 2.4. Statistical Analysis

All continuous variables are expressed in terms of mean ± standard deviation (SD) and range. Categorical variables are summarized in terms of absolute frequency and percentage. The Pearson correlation was used to explore relationships between age, HJHS, and HAL, while the Spearman analysis was used for the correlation between FISH and age and HJHS. All statistical analyses were considered significant for *p* < 0.05 and were performed using SPSS v.19.0 (IBM Corp., Armonk, NY, USA).

## 3. Results

Twenty patients, all male, with hemophilic arthropathy were enrolled according to the study criteria. Ten patients had severe hemophilia A, two had moderate hemophilia A, two mild hemophilia A, four severe hemophilia B, and two mild hemophilia B. The mean age of the patients was 40.63 ±10.54 years. For each patient, ten tendons were assessed, for a total number of 200 tendons evaluated.

### 3.1. Clinical–Functional Evaluation

The mean score of the HJHS scale was 14.1 points, with a high variance of 11.9. The FISH and HAL scores were 28.7 and 42.3, respectively. Age was correlated with HJHS and HAL (r = 0.507, *p* = 0.22; and r = −545, *p* = 0.13, respectively). This means that older patients had greater joint impairment and worse ability in activities. HJHS was also inversely correlated with FISH (r = −0.737, *p* < 0.0001), meaning that greater joint impairment corresponds to worse functional independence. From the values of the DASH scale, which is used in subjects with elbow involvement, impairment of upper limb function had a mean of 7.9%. As far as VISA-A is concerned, there was no difference between the right and left Achilles tendons, as the average score was 81.9 on the right and 83.9 on the left. In subjects who had joint involvement at the ankle, the VISA-A score was lower than 70, which is the cutoff for the presence of Achilles tendinopathy. The average score for VISA-P for the right and left patellar tendons was 69.6 and 68.8, respectively. In subjects with joint involvement at the knee, the VISA-P score was less than 60, which is the cutoff for the presence of patellar tendinopathy. The overall mean VAS score for pain was 2.2/10. The data are reported in Table 2.

### 3.2. Ultrasound Assessment

**Figure 1:**
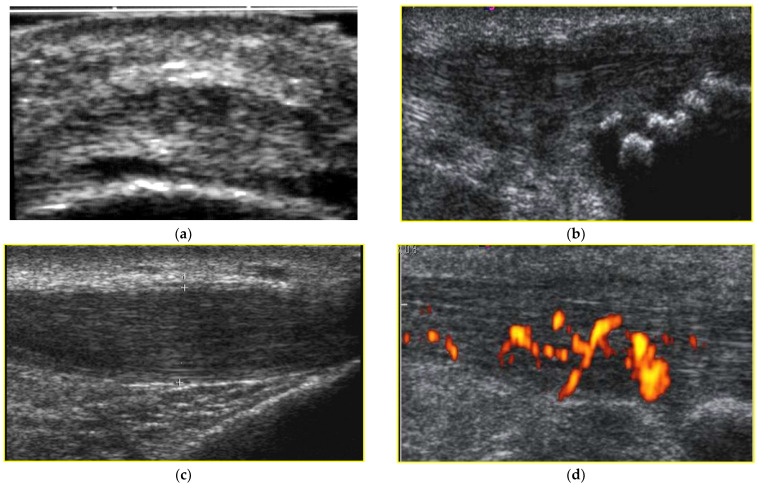
(**a**) Echostructural alterations (grade 2), (**b**) insertional tendon calcifications, (**c**) echostructural alterations (grade 1), and (**d**) signs of hyperemia on power color Doppler.

For the biceps tendon, there were no echostructural alterations or signs of hyperemia or neovascularization on the color Doppler and no intratendinous calcifications detected. As far as the triceps brachii tendon is concerned, one patient had grade-two echostructural changes in the left triceps tendon (Figure 1a), and one patient had grade-two neovascularization in the right triceps tendon. In one subject, intratendinous calcifications were present in both triceps tendons (Figure 1b). For the quadriceps tendon, grade-one echostructural changes were present in both quadriceps tendons in one subject, but there were no color Doppler signal changes in the quadriceps tendons of the studied subjects. Insertional tendon calcifications were present bilaterally in one subject. As regards the ultrasound parameters of the patellar tendon, no echostructural alterations were found, but there was the presence of a grade-one neovascularization signal with the color Doppler in a left patellar tendon. In one subject, there were intratendon calcifications bilaterally. For the Achilles tendon, in one subject, grade-one echostructural alterations were present in a right Achilles tendon (Figure 1c), and in another subject, grade-one positive color Doppler signals were present in a right Achilles tendon (Figure 1d). Insertional tendon calcifications were present bilaterally only in one subject. The data are reported in Table 3.

## 4. Discussion

Approximately 70% of the patients examined were affected by hemophilia A, in line with the prevalence of the disease in the general population compared to hemophilia B. From the results of the Hemophilia Joint Health Score (HJHS), the subjects, older at the time of evaluation, had a higher score than younger subjects regardless of the type of hemophilia. This indicates a greater involvement of joint damage linked to the underlying pathology, with a consequent reduction of the joint ROM at the level of the hemophilia target joints. This should be due to the fact that older patients followed an “on demand” treatment regimen for a longer time than younger subjects, that started before a regular prophylaxis, thus exposing the joints to repeated hemarthrosis and consequent joint damage. The high variation of SD of HJHS (11.9) and DASH (7.4) should be also due to the high difference of joint damages and functional limitations between younger and older patients and their different exposure to prophylaxis. There were no gender or race differences in the ultrasound evaluation of the tendons of the subjects in question because they were all Caucasian males. There is no study in the literature that performed the morphological study of tendons in hemophiliac patients; however, there is a little experience on the evaluation of tendon elasticity in patients with hemophilic arthropathy. There are only two studies by Cruz-Montecinos et al. in which greater stiffness and hysteresis of the Achilles tendon were highlighted in hemophiliac subjects compared to healthy subjects, combined with greater stiffness of the joints during the evaluation of the tendons during stretching exercises [13,14]. The purpose of this study was to evaluate the state of the satellite tendons of the target joints in patients with hemophilic arthropathy to search for alterations that may derive from direct damage of the disease and indirect damage due to alterations of the joint biomechanics. A set of morphological and echostructural parameters were selected on the basis of a systematic review and meta-analysis on the diagnosis of tendinopathy using ultrasound [15]. From the data collected for the tendon thickness, although there are no references in the literature for all tendons—being considered a non-specific parameter that is highly variable for age, sex, and race—no particular abnormalities were found for all the tendons assessed. The most specific parameter for diagnosing tendinopathy is the presence of signs of hyperemia and neovascularization on the color Doppler and the possible presence of intratendinous calcifications. In all of the tendons examined, echostructural alterations of a mild–moderate degree were identified in two subjects with moderate–severe joint damage. In three subjects with severe joint damage, the presence of color Doppler grade two in the right triceps tendon, grade one in the right Achilles tendon, and grade one in the left patellar tendon was identified. In one subject with severe diffuse joint damage, intratendinous calcifications were present in all tendons studied, except the biceps tendon. The tendons had a reduced vascular supply compared to the muscles to which they are connected. Nonetheless, the presence of vessels in the tendons is very important for the normal function of the tenocytes and the reparative capacity of the tendon itself. The blood supply to the tendon is ensured, to a small extent, by the vessels coming from the muscle belly and from the periosteum surrounding the osteotendinous junction and, for the rest, by the vascular network of the peritendinous sheets and the synovial sheath in the sites where this is present [25]. In hemophiliac patients, intratendinous bleeding was not detected. It can be hypothesized that tendon alterations in the evaluated patients were not related to specific damage to tendons due to the underlying pathology but were probably consequent to the joint damage, with a reduction of the physiological joint ROM and rigidity. The finding of the absence of specific tendon abnormalities due to bleeding at this level allows us to introduce some rehabilitative considerations [26,27,28]. During rehabilitation treatment, the prevention of joint stiffness and consequent reduction of the biomechanical stress to which the tendons of the target joints of hemophilic arthropathy are subjected are one of the most important goals to reduce disability in joint function [29,30,31]. Soft stretching and eccentric exercises are generally proposed as effective treatments in managing patients suffering from tendinopathies [32,33,34]. It has been shown that such exercises promote the formation of cross-link collagen fibers and facilitate tendon remodeling. It is hypothesized that eccentric exercise promotes a structural adaptation of the musculotendon unit in order to protect it from excessive stress. [35,36] The basic principles of an eccentric exercise program must take into account the length of the tendon, the load, and the speed of execution of the exercise. If the tendon is pre-stretched, its length at rest will be greater and will therefore be less stretched during movement. [37,38]. In the absence of evidence of tendon damage due to the hemophilia, these techniques could be safely introduced to counteract joint stiffness and muscle hypotrophy [39,40]. However, the tendons in the chronic phase should be re-evaluated to monitor signs of bleeding and remodulate the rehabilitation treatment.

## 5. Conclusions

In conclusion, the tendons of the target joints in the patient with hemophilic arthropathy are likely not directly compromised by the underlying disease but by the indirect biomechanical damage caused by the hemophilic joint disease. For this reason, rehabilitation treatment with eccentric exercises can be considered safe and effective in improving the tenso-elastic properties of the tendon and making it more resistant to mechanical stress [32,34]. Future studies will be necessary to compare these results, since the sample of data collected was small due to the low number of hemophiliac subjects in the general population, and to identify the possible presence of echostructural alterations at the tendon level to establish adequate rehabilitation treatment.

## Figures and Tables

**Table 1:** Ultrasound classification.

Echostructural Alteration	Signs of Hyperemia on Power-Color Doppler
Grade 0—normal tendon	Grade 0—no visible new vessels
Grade 1—slight echostructural alteration	Grade 1—one to two new vessels
Grade 2—moderate echostructural alteration	Grade 2—few vessels/low blood flow
Grade 3—marked echo structural alteration and degeneration	Grade 3—many vessels/important blood flow

**Table 2:** Rating scales.

Rating Scale	Mean	SD
HJHS score	14.1	11.9
HAL score	42.3	23.7
FISH score	28.7	3.9
DASH score	7.9	7.4
VAS score	2.2	2.1
VISA-A right	69.6	14.8
VISA-A left	68.8	15.4
VISA-P right	81.9	16.6
VISA-P left	83.9	17.2

**Table 3:** Ultrasound parameters.

	Bicipital Tendon		Tricipital Tendon		Patellar Tendon		Quadricipital Tendon		Achilles Tendon	
	Mean	SD	Mean	SD	Mean	SD	Mean	SD	Mean	SD
Tendon thickness—right (mm)	4.2	0.6	4.1	0.4	4.2	0.6	4.8	1.1	4.3	0.5
Tendon thickness—left (mm)	4.0	0.6	4.0	0.3	4.0	0.6	4.8	1.1	4.3	0.4
Echogenicity—right	0	0	0	0	0	0	0.05	0.2	0.05	0.2
Echogenicity—left	0	0	0.1	0.4	0	0	0.05	0.2	0	0
Signs of hyperemia on P.D.—right	0	0	0.1	0.4	0	0	0	0	0.05	0.2
Signs of hyperemia on P.D.—left	0.05	0.2	0	0	0.05	0.22	0	0	0	0
Calcifications—right	0.05	0.2	0.05	0.2	0.05	0.22	0.05	0.2	0.05	0.2
Calcifications—left	0.05	0.2	0.05	0.2	0.05	0.22	0.05	0.2	0.05	0.2

## Data Availability

The data that support the findings of this study are available from the corresponding author upon reasonable request.

## References

[1] Roosendaal G., Lafeber F.P. (2006). Pathogenesis of haemophilic arthropathy. Haemophilia.

[2] Dunn A.L. (2011). Pathophysiology, diagnosis and prevention of arthropathy in patients with haemophilia. Haemophilia.

[3] Srivastava A., Brewer A.K., Mauser-Bunschoten E.P., Key N.S., Kitchen S., Llinas A., Ludlam C.A., Mahlangu J.N., Mulder K., Poon M.C. (2013). Guidelines for the management of hemophilia. Haemophilia.

[4] Lafeber F.P., Miossec P., Valentino L.A. (2008). Physiopathology of haemophilic arthropathy. Haemophilia.

[5] De la Corte-Rodriguez H., Rodriguez-Merchan E.C., Jimenez-Yuste V. (2018). Point-of-care Ultrasonography in Orthopedic Management of Hemophilia: Multiple Uses of an Effective Tool. HSSJ.

[6] Kidder W., Nguyen S., Larios J., Bergstrom J., Ceponis A., von Drygalski A. (2015). Point-of-care musculoskeletal ultrasound is critical for the diagnosis of hemarthroses, inflammation and soft tissue abnormalities in adult patients with painful haemophilic arthropathy. Haemophilia.

[7] Muc M., Perja A., Riva S., Grochowska B., Mangiafico L., Mago D., Gringeri A. (2012). Ultrasonography of haemophilic arthropathy. Haemophilia.

[8] Martinoli C., Della Casa Alberighi O., Di Minno G., Graziano E., Molinari A.C., Pasta G., Russo G., Santagostino E., Tagliaferri A., Tagliafico A. (2013). Development and definition of a simplified scanning procedure and scoring method for Haemophilia Early Arthropathy Detection with Ultrasound (HEAD-US). Thromb. Haemost..

[9] Martinoli C., Di Minno M.N.D., Pasta G., Tagliafico A. (2016). Point-of-care ultrasound in haemophilic arthropathy: Will the HEAD-US system supplement or replace physical examination?. Haemophilia.

[10] Querol F., Rodriguez-Merchan E.C. (2012). The role of ultrasonography in the diagnosis of the musculoskeletal problems of haemophilia. Haemophilia.

[11] Rodriguez-Merchan E.C. (2010). Musculoskeletal Complications of Hemophilia. HSSJ.

[12] Hanley J., McKernan A., Creagh M.D., Classey S., McLaughlin P., Goddard N., Briggs P.J., Frostick S., Giangrande P., Wilde J. (2017). Guidelines for the management of acute joint bleeds and chronic synovitis in haemophilia. Haemophilia.

[13] Cruz-Montecinos C., Pérez-Alenda S., Oyarzún-Tejeda A., Cerda M., Querol-Fuentes F. (2015). Estimation of tensile properties of the Achilles tendon in haemophilic arthropathy of the ankle: Case study. Haemophilia.

[14] Cruz-Montecinos C., Pérez-Alenda S., Contreras-Sepúlveda F., Querol F., Cerda M., Maas H. (2019). Assessment of tensile mechanical properties of the Achilles tendon in adult patients with haemophilic arthropathy. Reprod. Study Haemoph..

[15] Matthews W., Ellis R., Furness J., Hing W. (2018). Classification of Tendon Matrix Change Using Ultrasound Imaging: A Systematic Review and Meta-analysis Review. Ultrasound Med. Biol..

[16] Hilliard P., Funk S., Zourikian N., Bergstrom B.-M., Bradley C.S., McLimont M., Manco-Johnson M., Petrini P., Berg M.V.D., Feldman B.M. (2006). Hemophilia joint health score reliability study. Haemophilia.

[17] St-Louis J., Audrey A., Funk S., Tilak M., Classey S., Zourikian N., McLaughlin P., Lobet S., Hernandez G., Akins S. (2022). Hemophilia Joint Health Score version 2.1 Validation in Adult Patients Study: A multicenter international study. Res. Pr. Thromb. Haemost..

[18] Van Genderen F.R., Westers P., Heijnen L., Kleijn P., Berg H.M., Helders P.J.M., Meeteren N.L.U. (2006). Measuring patients’ perceptions on their functional abilities: Validation of the Haemophilia Activities List. Haemophilia.

[19] Poonnoose P.M., Manigandan C., Thomas R., Shyamkumar N.K., Kavitha M.L., Bhattacharji S., Srivastava A. (2005). Functional independence score in haemophilia (FISH): A new performance-based instrument to measure disability. Haemophilia.

[20] Waterfield J., Sim J. (1996). Clinical assessment of pain by the visual analogue scale. Br. J. Ther. Rehabil..

[21] Roach K.E., Budiman-Mak E., Songsiridej N., Lertratanakul Y. (1991). Development of a shoulder pain and disability index. Arthritis Rheum. Off. J. Am. Coll. Rheumatol..

[22] Gummesson C., Atroshi I., Ekdahl C. (2003). The disabilities of the arm, shoulder and hand (DASH) outcome questionnaire: Longitudinal construct validity and measuring self-rated health change after surgery. BMC Musculoskelet. Disord..

[23] Maffulli N., Umile G.L., Testa V., Oliva F., Capasso G., Denaro V. (2008). Italian translation of the VISA-A score for tendinopathy of the main body of the Achilles tendon. Disabil. Rehabil..

[24] Maffulli N., Umile G.L., Testa V., Oliva F., Capasso G., Denaro V. (2008). VISA-P score for patellar tendinopathy in males: Adaptation to Italian. Disabil. Rehabil..

[25] Schatzker J., Branemark P.I. (1969). Intravital observations on the microvascular anatomy and microcirculation of the tendon. Acta Orthop. Scand..

[26] Cuesta-Barriuso R., Gómez-Conesa A., López-Pina J.A. (2013). Physiotherapy treatment in patients with hemophilia and chronic ankle arthropathy: A systematic review. Rehabil. Res. Pr..

[27] De la Corte-Rodriguez H., Rodriguez-Merchan E.C. (2013). The role of physical medicine and rehabilitation in haemophiliac patients. Blood Coagul. Fibrinolysis.

[28] Hilberg T., Herbsleb M., Gabriel H.H.W., Jeschke D., Schramm W. (2001). Proprioception and isometric muscular strength in haemophilic subjects. Haemophilia.

[29] Koch B., Luban N.L., Galioto F.M., Rick M.E., Goldstein D., Kelleher J.F. (1984). Changes in coagulation parameters with exercise in patients with classic hemophilia. Am. J. Hematol..

[30] Poonnoose P.M., Srivastava A. (2006). Functional assessment of arthropathy-An international perspective. Semin. Hematol..

[31] Rodriguez-Merchan E.C. (1999). Therapeutic options in the management of articular contractures in haemophiliacs. Haemophilia.

[32] Garau G., Oliva F., Longo G.U., Maffulli N., Oliva F. (2010). Conservative management of Achilles tendinopathy: Eccentric exercises. Achilles Tendon. Tecniche Chirurgiche in Ortopedia e Traumatologia.

[33] Schafer G.S., Valderramas S., Gomes A.R., Budib M.B., Wolff L.P., Ramos A.A.T. (2016). Physical exercise, pain and musculoskeletal function in patients with haemophilia: A systematic review. Haemophilia.

[34] Stanish W.D., Rubinovich R.M., Curwin S. (1986). Eccentric exercise in chronic tendinitis. Clin. Orthop. Relat. Res..

[35] Stephensen D., Bladen M., McLaughlin P. (2018). Recent advances in musculoskeletal physiotherapy for haemophilia. Ther. Adv. Hematol..

[36] Strike K., Mulder K., Michael R. (2016). Exercise for haemophilia. Cochrane Database Syst. Rev..

[37] Van Genderen F.R., Fischer K., Heijnen L., Kleijn P., Berg H.M.V.D., Helders P.J.M., Meeteren N.L.U. (2006). Pain and functional limitations in patients with severe haemophilia. Haemophilia.

[38] Wallny T., Brackmann H., Kraft C., Nicolay C., Pennekamp P. (2006). Achilles tendon lengthening for ankle equinus deformity in hemophiliacs. Acta Orthop..

[39] Wells A.J., Stephensen D. (2020). The role of the physiotherapist in the management of people with haemophilia: Defining the new normal. Br. J. Hosp. Med..

[40] Scaturro D., Benedetti M.G., Lomonaco G., Tomasello S., Farella G.M., Frizziero A., Mauro G.L. (2021). Effectiveness of rehabilitation on pain and function in people affected by hemophilia. Medicine.

